# mTOR regulates the expression of DNA damage response enzymes in long‐lived Snell dwarf, GHRKO, and PAPPA‐KO mice

**DOI:** 10.1111/acel.12525

**Published:** 2016-09-13

**Authors:** Graham Dominick, Jacqueline Bowman, Xinna Li, Richard A. Miller, Gonzalo G. Garcia

**Affiliations:** ^1^ Department of Molecular, Cellular, and Developmental BiologyUniversity of Michigan College of Literature, Science and the ArtsAnn ArborMIUSA; ^2^ Department of PathologyUniversity of Michigan School of MedicineAnn ArborMIUSA; ^3^ University of Michigan Geriatrics CenterAnn ArborMIUSA

**Keywords:** DNA repair, gene expression, IGF, molecular biology of aging, signal transduction

## Abstract

Studies of the mTOR pathway have prompted speculation that diminished mTOR complex‐1 (mTORC1) function may be involved in controlling the aging process. Our previous studies have shown diminished mTORC1 activity in tissues of three long‐lived mutant mice: Snell dwarf mice, growth hormone receptor gene disrupted mice (GHRKO), and in this article, mice deficient in the pregnancy‐associated protein‐A (PAPPA‐KO). The ways in which lower mTOR signals slow aging and age‐related diseases are, however, not well characterized. Here, we show that Snell, GHKRO, and PAPPA‐KO mice express high levels of two proteins involved in DNA repair, O‐6‐methylguanine‐DNA methyltransferase (MGMT) and N‐myc downstream‐regulated gene 1 (NDRG1). Furthermore, we report that lowering mTOR enhances MGMT and NDRG1 protein expression via post‐transcriptional mechanisms. We show that the CCR4‐NOT complex, a post‐transcriptional regulator of gene expression, is downstream of the mTORC1 pathway and may be responsible for the upregulation of MGMT and NDRG1 in all three varieties of long‐lived mice. Our data thus suggest a novel link between DNA repair and mTOR signaling via post‐transcriptional regulation involving specific alteration in the CCR4‐NOT complex, whose modulation could control multiple aspects of the aging process.

**Abbreviations**
DW: Snell dwarf mice
GHRKO: growth hormone receptor deletion mice
MGMT: O‐6‐methylguanine‐DNA methyltransferase
mTOR: mammalian target of rapamycin
NDRG1: N‐myc downstream‐regulated gene 1
PAPPA‐KO or KO: pregnancy‐associated protein‐A deletion mice

## Introduction

Several lines of evidence have implicated the mechanistic target of rapamycin (mTOR) in the regulation of aging. In lower organisms, such as *Caenorhabditis elegans* and *Drosophila melanogaster*, inactivation or reduction of mTOR activity results in life extension (Sharp *et al*., 2013). In addition, reduction of mTOR activity by rapamycin (Harrison *et al*., 2009) or reduction in expression of mTOR kinase by hypomorphic mTOR alleles (Selman *et al*., 2009; Lamming *et al*., 2012; Wu *et al*., 2013) can increase mouse lifespan. mTOR is a serine/threonine kinase that forms the catalytic core of at least two complexes, mTOR complex‐1 (mTORC1) and mTOR complex‐2 (mTORC2). *In vivo*, the phosphorylation of ribosomal protein S6 (pS6_(235)_) and initiation factor 4E‐binding protein (p4E‐BP1_(37)_) are markers of mTORC1 activity. Phosphorylation of AKT at Ser‐473 (pAKT_(473)_), Ser‐450 (pAKT_(450)_), and the N‐myc downstream‐regulated gene (pNDRG_(346)_) are considered markers of mTORC2 activity. We have recently studied the phosphorylation status of these substrates in the Snell and GHRKO mice, two long‐lived genetic models, and found tissue‐specific reduction in mTORC1 activity as well as alteration in mTORC2 activity (Dominick *et al*., 2015). Interestingly, neither mTORC1 nor mTORC2 was altered in mice with liver‐specific deletion of GHR, mice in which lifespan is normal despite dramatically lower levels of serum IGF‐1 (Bartke, 2008; Dominick *et al*., 2015). The metalloproteinase PAPP‐A is thought to act by regulating local IGF‐1 action through degradation of some IGF‐1 binding proteins, particularly IGF‐BP4 (Conover & Bale, 2007). PAPP‐A deletion can extend both mean and maximum lifespan by 20–40%, with a reduced incidence of spontaneous tumors, without modulation of serum levels of GH or IGF‐1. Effects of PAPPA‐KO on mTORC1 and mTORC2 have not yet been reported. The mechanistic links between GH/IGF‐1 regulation and prolongation of lifespan in these three varieties of mice are not well understood.

O‐6‐methylguanine‐DNA methyltransferase (MGMT) is an enzyme involved in the direct reversal of DNA damage *in vitro* and postmitotic cells *in vivo* by methylating agents used clinically for chemotherapy (Iyama & Wilson, 2013; Jiang *et al*., 2014). It have been suggested that MGMT may play a role in regulating mouse lifespan (Meira *et al*., 2014), but it is not known whether long‐lived mice, such as Snell, GHRKO, and PAPPA‐KO, have altered expression of MGMT. Higher expression of NDRG1, a protein of unknown function, can promote MGMT protein stability and activity in human glioma cells by mechanisms that include protein interactions and preferential translation (Smalley *et al*., 2014; Weiler *et al*., 2014). Therefore, understanding the signaling pathways regulating MGMT and NDRG1 may provide insights into resistance to DNA damage, chemotherapy, and the aging process. We hypothesized that higher expression of MGMT and NDRG1 may be a common feature shared by Snell, GHRKO, and PAPPA‐KO mice, and that mTOR activity could be a key upstream regulator of MGMT and NDRG1 expression. Microarray analysis of the mRNA gene expression profiles of many different models of slow‐aging mice have not led to a consensus on common pathway(s) central to the aging process, including mice whose lifespan has been increased by mTOR reduction by drugs (Yu *et al*., 2015) or dietary interventions (Swindell, 2008). However, it is plausible that improved DNA repair may be one important common feature (Meira *et al*., 2014). Discrepancy between mRNA levels and protein expression in many genes complicates interpretation of mRNA patterns in tissues of slow‐aging mice, and it is likely that gene regulation by post‐transcriptional mechanisms, including changes in mRNA stability (Brewer, 2002; Lee & Gorospe, 2010), regulation by microRNAs (Bates *et al*., 2010; Liang *et al*., 2011), and sequestration to P and ER bodies may contribute to altered levels of DNA repair proteins in slow‐aging mice.

The CCR4‐NOT complex is a key regulator of mRNA stability, microRNA function, and preferential translation, and functions as a check point in the control of translation and protein degradation (Inada & Makino, 2014; Shirai *et al*., 2014). The CCR4‐NOT complex is comprised of at least ten subunits, but there is limited information about the function of individual components of the complex in mammals. Recent reports using *in vitro* methods have helped to clarify some functional roles of CNOT components (Laribee *et al*., 2015; Okada *et al*., 2015; Rodriguez‐Gil *et al*., 2016). Mice in which CNOT6 has been deleted are viable but, with exception of some bone abnormalities, do not have obvious changes in phenotype. However, CNOT3 heterozygous mice (Morita *et al*., 2011) display some phenotypes reminiscent of Snell (Bartke & Brown‐Borg, 2004; Boylston *et al*., 2006), Ames dwarf (Boylston *et al*., 2006), GHRKO (Brown‐Borg & Bartke, 2012), and PAPPA‐KO mice (Hill *et al*., 2015), including higher insulin sensitivity, improved glucose tolerance, and higher oxygen consumption. Here, we present evidence suggesting that Snell, GHRKO, and PAPPA‐KO mice express high levels of NDRG1 and MGMT proteins that are under the control of mTORC1 activity, and that this regulation involves post‐transcriptional mechanisms that may depend on alteration of the CCR4‐NOT complex.

## Results

### Multiple models of slow aging, including PAPPA‐KO mice, show lower mTORC1 activity

**Figure 1:**
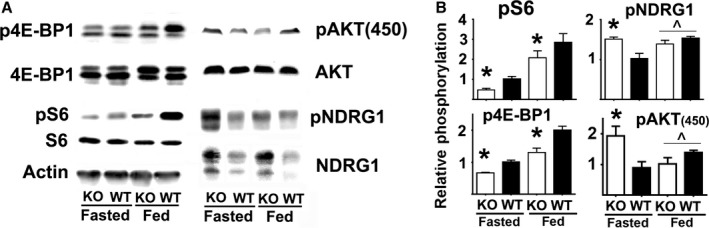
mTORC activity in liver of PAPP‐A mice. (A) Representative Western blots showing the phosphorylation of S6, 4E‐BP1, AKT, and NDRG1 in liver samples from PAPPA‐KO (KO) and control littermates (WT) under fasting (Fasted) or fed (Fed) conditions. (B) Quantification of pS6, p4E‐BP1, pAKT(450), and pNDRG1 phosphorylation as ratios to the level of the corresponding protein. All bar graphs represent the mean ± SEM from a total of eight mice (four females and four males) per group and are normalized with respect to ratio of 1 in fasted WT for each protein. The asterisk (*) indicates statistical significance relative to the fasting WT from each group.

We have recently reported that Snell and GHRKO mice show reduced mTORC1 activity in multiple tissues, accompanied by changes in both fasting and food‐induced mTORC2 activity (Dominick *et al*., 2015). To see whether PAPPA‐KO mice showed similar changes in mTORC1 and mTORC2 activity, we measured phosphorylation of mTORC1 substrates pS6_(S235/236)_ [referred to as pS6 in figures and text], p4E‐BP1_(T37/46)_ [termed p4E‐BP1 in figures and text] and mTORC2 substrates pAKT_(T450)_ and pNDRG1_(T346)_ [termed pNDRG1 in figures and text], either after 18 h of fasting or after 18 h of fasting followed by 6 h of access to food (Dominick *et al*., 2015). Figure 1A shows a representative Western blot for liver, and Fig. 1B shows mean values from three independent experiments using at least eight mice per group. We found no significant sex effects (half of the mice were males) on any of the measures reported in this article (data not shown). Table S1 shows parallel experiments for muscle, heart, and kidney of PAPPA‐KO mice for the mTORC1 substrates, with estimates of fold induction as well as the results of ANOVA testing significance of the effects of genotype (G), nutritional conditions (fasting vs. feeding, N), and GxN interactions (Int). We found that PAPPA‐KO mice show lower levels of pS6 and p4E‐BP1, independent of nutritional status, in all four tissues tested (Fig. 1 and Table S1, Supporting information). Two‐factor ANOVA confirmed the significant effect of the PAPP‐A deletion (genotype effects: WT > KO) and feeding (nutritional effects: fed > fasting), without evidence for interaction. The result is similar to our findings for Snell and GHRKO mice (Dominick *et al*., 2015). We found no differences in the expression of total S6 or 4E‐BP protein in any of the groups (data not shown).

Results for mTORC2 substrates pAKT_(450)_ and pNDRG are shown in Fig. 1 for liver and are summarized in Table S2 (Supporting information) for all four tissues. Consistent with Snell and GHRKO mice (Dominick *et al*., 2015), fasted PAPPA‐KO mice have higher mTORC2 activity, and this activity decreases in response to feeding in PAPPA‐KO but not in WT control mice (Fig. 1). Statistical analysis using ANOVA confirmed that feeding has significant interaction effects for pAKT_(450)_ and pNDRG (Fig. 1B and Table S2, Supporting information). The overall results suggest that PAPPA‐KO, Snell, and GHRKO mice share a common regulatory mechanism for mTOR activity and signaling that includes tissue‐specific declines in mTORC1 activity and differential regulation of mTORC2 activity.

### Snell, GHRKO, and PAPPA‐KO mice show higher NDRG1 and MGMT protein expression, but no changes in NDRG1 or MGMT mRNA expression

**Figure 2:**
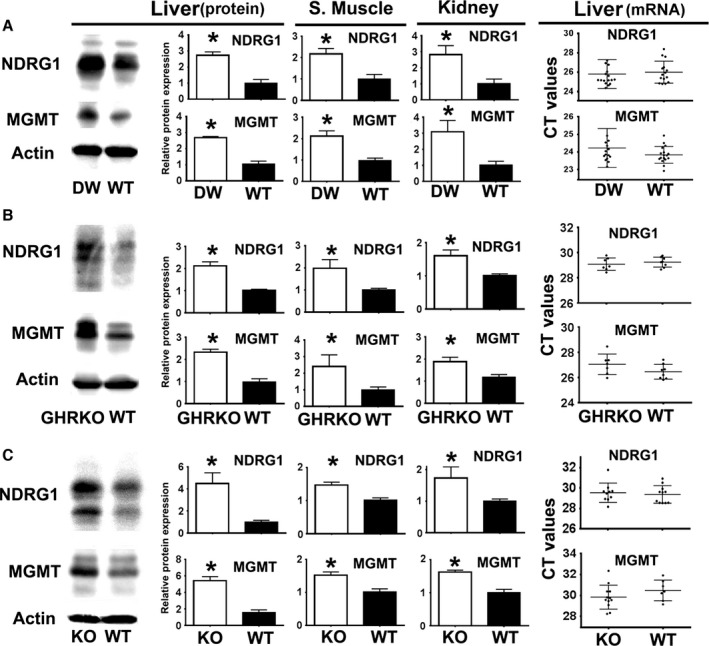
Snell mice (DW), GHRKO, and PAPP‐A (KO) mice show higher NDRG1 and MGMT protein expression but no significant change in mRNA expression. (A) Representative Western blots showing NDRG1 and MGMT expression in Snell mice (DW) from liver (left). Quantification in liver, skeletal muscle, and kidney (middle panels) is shown as mean ± SEM from three experiments, using a total of at least 12 mice (six females and six males) per group, using normalization as described in Fig. 1. The qRT–PCR CT dotplot values for NDRG1 and MGMT expression are shown in the right panels. Each symbol represents an individual mouse. Horizontal lines show mean ± SEM from a total of 16 mice (eight females and eight males) per group. The asterisk (*) indicates statistical significance relative to the WT controls of each group. (B) Results for GHRKO mice from at least 11 mice (six females and five males) per group for protein expression (middle panels) and for qRT–PCR using eight mice (four females and four males) per group (right panels). (C) Results for PAPPA‐KO (KO) mice from at least eight mice (four females and four males) per group and qRT–PCR using at least ten mice (five females and five males) per group.

Using a rabbit monoclonal from Cell Signaling (D6C2), we noted an increase in levels of total NDRG1 protein expression in liver of PAPPA‐KO mice (Fig. 1A). Higher NDRG1 expression has been associated with enhanced DNA repair through increased MGMT protein stability/activity (Weiler *et al*., 2014). We therefore measured NDRG1 and MGMT protein expression in liver, skeletal muscle (upper leg), and kidney from our three slow‐aging mice models fed ad lib. The right panels of Fig. 2 show representative results from liver tissue in Snell (Fig. 2A), GHRKO (Fig. 2B), and PAPPA‐KO (Fig. 2C). NDRG1 and MGMT protein expression was increased by 2× to 3× in each kind of slow‐aging mouse.

To assess whether higher protein levels were the result of upregulation of NDRG1 and MGMT mRNA transcripts, we performed qRT–PCR for both genes in liver. In contrast to the protein data, there were no differences in the levels of NDRG1 and MGMT mRNA in any of the mouse models (Fig. 2 right panels). We confirmed these results using three other differently designed probes for MGMT and NDRG1 (data not shown). We also found no differences in the GAPDH mRNA expression (Fig. S1, Supporting information). This suggested that discrepancies between protein expression and mRNA levels might reflect differential post‐transcriptional regulation of MGMT and NDRG1 in slow‐aging mice.

### mTOR signaling regulates NDRG1 and MGMT protein expression but not their mRNA expression

**Figure 3:**
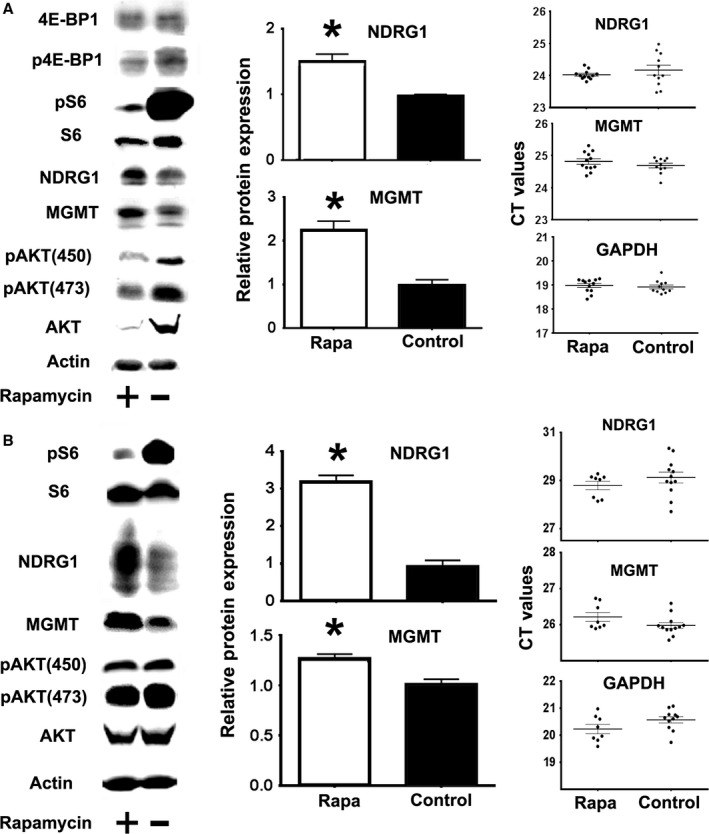
Rapamycin enhances MGMT and NDRG1 protein expression but does not significantly alter their mRNA expression. (A) Representative Western blots showing pS6, p4E‐BP1, and pAKT phosphorylation (in parallel to total S6, 4E‐BP1 and AKT protein), as well as NDRG1 and MGMT expression in mouse fibroblasts, either untreated (−, Control) or treated with rapamycin (+, Rapa). Bar graphs represent the mean ± SEM from six independent experiments normalized with respect to WT controls. The CT value dotplots show mRNA levels from six independent experiments. The (*) indicates statistical significance. (B) Analysis of rapamycin effects in liver of UM‐HET mice from at least eight mice per group.

In some human cell lines, rapamycin (an mTOR inhibitor) can enhance MGMT protein expression, and this increase is the result of preferential translation of MGMT transcripts (Smalley *et al*., 2014). mTOR can also regulate stress resistance by preferential translation of certain mRNAs (Reiter *et al*., 2004), including ATF‐4 (Ait *et al*., 2012). Hence, we hypothesized that changes in mTOR function in Snell, GHRKO, and PAPPA‐KO mice could increase NDRG1 and MGMT via a post‐transcriptional pathway. To test this idea, we treated mouse fibroblasts from a genetically heterogeneous stock of mice (Harrison *et al*., 2009) with rapamycin for 48 h and analyzed the expression of NDRG1 and MGMT using Western blots and qRT–PCR. The data (Fig. 3) show that rapamycin enhances NDRG1 and MGMT protein expression, at a concentration that impairs phosphorylation of the mTORC1 substrates 4E‐BP1 and S6. Statistical analysis confirmed significant increases in NDRG1 and MGMT protein in fibroblasts treated for 48 h with rapamycin (Fig. 3A middle panel). Parallel mRNA analysis found no differences in NDRG1 and MGMT mRNA expression (or in GAPDH mRNA) in fibroblasts exposed to rapamycin for 24 or 72 h (not shown). These results suggest that mTOR regulates fibroblast NDRG1 and MGMT protein expression through a post‐transcriptional mechanism. However, extended rapamycin treatments can diminish expression and phosphorylation of some of the mTORC2 substrates [pAKT/AKT in Fig. 3A and (Sarbassov *et al*., 2006; Lamming *et al*., 2012)], suggesting that lower mTORC2 function may, in part, be responsible for some of the effects we see in MGMT and NDRG1 expression. The overall results suggest that mTOR plays a key role, but more work will be needed to determine the specific role of each complex (mTORC1 and mTORC2) in these animals.

We also tested the *in vivo* effects of rapamycin on NDRG1 and MGMT in liver. A single rapamycin injection increases NDRG1 and MGMT protein expression in liver (Fig. 3B). Rapamycin leads to the expected reduction in mTORC1 activity (indicated by pS6), and no apparent changes in mTORC2 activity (as measured by phosphorylation of AKT_450_ and AKT_473_). Statistical analysis showed significant increases in NDRG1 and MGMT protein expression by rapamycin. Consistent with the fibroblast experiments, there are no significant changes in NDRG1, MGMT mRNA levels. The *in vivo* results suggest that mTOR can regulate NDRG1 and MGMT expression through post‐transcriptional mechanisms, and that lower mTORC1 activity in Snell, GHRKO, and PAPPA‐KO mice may upregulate MGMT and NDRG1. However, Schreiber *et al*. (2015) have suggested that rapamycin effects on mTORC2 signaling may be difficult to detect, and we cannot rule out the idea that mTORC2 could also play an important role in regulating MGMT and NDRG1 expression in the slow‐aging mice.

### Snell, GHRKO, and PAPPA‐KO mice show similar alterations in the expression of components of the CCR4‐NOT complex

**Figure 4:**
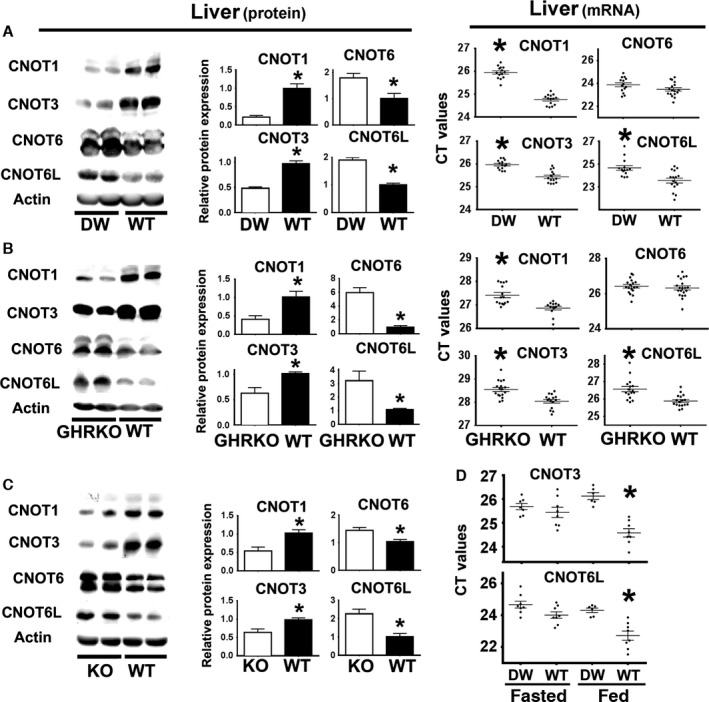
Livers of Snell mice (DW), GHRKO, and PAPPA‐KO (KO) mice show declines in CNOT1 and CNOT3 accompanied by increases in CNOT6L and CNOT6 protein. (A) Left panels represent Western blots of CCR4‐CNOT complex components in Snell mice (DW) and control mice (WT) from liver. Quantification shown as mean ± SEM of three experiments in middle panels from a total of 12 mice (six females and six males) per group using normalization as described in Fig. 1. Right panel shows the qRT–PCR CT values for CCR4‐CNOT mRNA levels in livers from at least 14 mice (seven females and seven males) per group. The (*) indicates statistical significance relative to the WT controls of each group. (B) Data for protein levels in GHRKO mice (left and middle panels), using six mice (three females and three males) per group. qRT–PCR CT data (right panels) from at least 12 mice (six females and six males) per group. (C) Data for protein in PAPPA‐KO (KO) mice, using nine mice (five females and four males) per group. (D) Effect of fasting and feeding on mRNA expression of CNOT3 and CNOT6L in livers of Snell (DW) and WT control mice from at least six mice (1/2 females and 1/2 males) per group. The (*) indicates statistical significance relative to Snell (DW).

To see whether changes in components of the CCR4‐NOT complex could be involved in differential post‐transcriptional regulation, we tested expression of CNOT1, CNOT3, CNOT6, and CNOT6L in liver of Snell, GHRKO, and PAPPA‐KO mice. CNOT3 was selected because its decline leads to some features shared with slow‐aging mice (Morita *et al*., 2011). CNOT6 and CNOT6L were tested because they may regulate DNA repair (Sanchez‐Perez *et al*., 2009). As shown in Fig. 4, CNOT1 and CNOT3 levels are lower, and CNOT6 and CNOT6L higher, in liver from each variety of slow‐aging mice.

CNOT1 and CNOT3 mRNA CT values are higher in Snell and GHRKO liver (Fig. 4) in good agreement with the protein data. In contrast, we found no changes in the CNOT6 mRNA levels with respect to WT controls and, unexpectedly, found a significant reduction in CNOT6L mRNA (Fig. 4A,B). The results of CNOT6 and CNOT6L do not correspond with the level of their protein expression. We do not know the reason for this discrepancy but it suggests that CNOT components may also themselves be subject to post‐transcriptional regulation. CNOT4, which does not associate with CCR4‐NOT in mammals and does not regulate mRNA stability or translation (Shirai *et al*., 2014), did not differ between long‐lived and littermate control mice (Fig. S2, Supporting information).

Feeding mice increases CNOT3 expression (Morita *et al*., 2011). Interesting, provision of food after a period of fasting increased CNOT3 and CNOT6L mRNA expression in control mice, but not in Snell littermates (Fig. 4D). These results could be explained by a higher level of mTORC1 activity in control mice (WT) that could contribute to the differential upregulation of CNOT3 and CNOT6L mRNA in response to food.

### Rapamycin inhibition of mTORC1 activity mimics some of the changes in the CCR4‐CNOT complex expression found in Snell, GHRKO, and PAPPA‐KO mice

**Figure 5:**
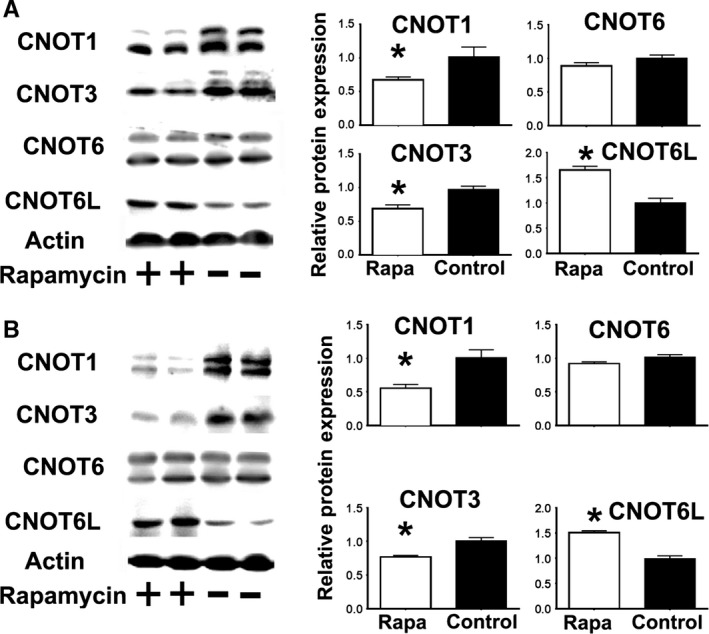
Rapamycin treatment decreases CNOT1 and CNOT3 expression and increases CNOT6L protein expression, but does not affect CNOT6 expression. (A) Representative Western blots showing expression of components of the CCR4‐CNOT complex in mouse fibroblasts untreated (−, Control) or treated with rapamycin (+, Rapa). Bar graphs represent the mean ± SEM from a total of six independent experiments normalized with respect to WT controls. The (*) indicates statistical significance. (B) Analysis of rapamycin effects on CNOT components in mouse livers, either untreated (−, Control) or treated with rapamycin (+, Rapa) from at least eight mice per group (50% males).

To test the hypothesis that mTOR can regulate the expression of components of the CCR4‐NOT complex, we treated primary mouse fibroblasts with rapamycin and analyzed the expression of CNOT1, CNOT3, CNOT6, and CNOT6L using Western blots and qRT–PCR. Rapamycin significantly decreased CNOT1 and CNOT3 protein expression and enhanced expression of CNOT6L; however, CNOT6 protein was not affected (Fig. 5A). Rapamycin significantly diminished CNOT1, CNOT3, and CNOT6L mRNA but not CNOT6 mRNA (Fig. S3, Supporting information). This pattern of consistent changes in protein and mRNA data for CNOT1, CNOT3, and CNOT6L is similar to that noted for Snell, GHRKO, and PAPPA‐KO mice (Fig. 4). For *in vivo* confirmation, we treated mice with rapamycin for 48 h and analyzed the expression of CCR4‐NOT components in liver. Rapamycin reduced CNOT1 and CNOT3 and enhanced CNOT6L, but did not change CNOT6 (Fig. 5B), consistent with the fibroblast results. Thus, CNOT1, CNOT3, and CNOT6L are under the control of mTOR signaling at the level of transcription and post‐transcriptional mechanisms. In contrast, CNOT6 is not affected by rapamycin at the dose and time used.

### CNOT3 regulates NDRG1 and MGMT protein expression

**Figure 6:**
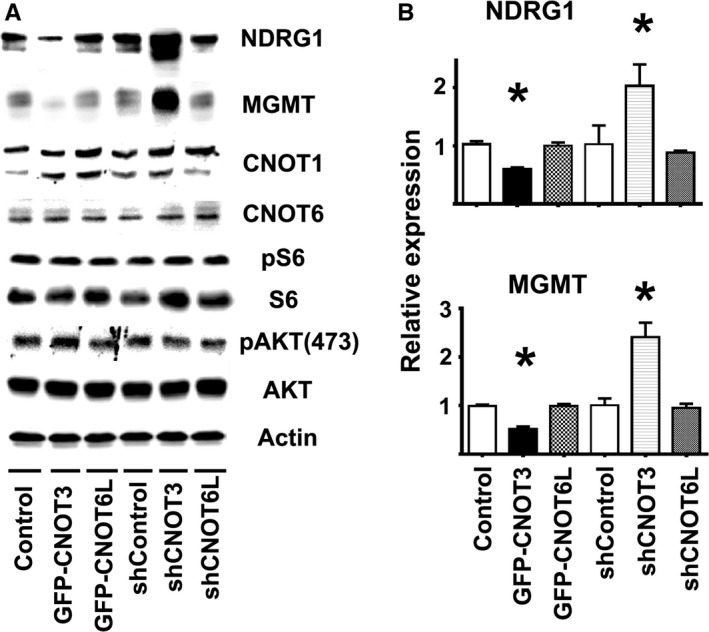
Effect of overexpressing or downregulating CNOT3 and CNOT6L protein on NDRG1 and MGMT protein expression in mouse fibroblasts. (A) Representative Western blots for NDRG1 and MGMT expression after 72 h of transfection, together with pS6, pAKT, CNOT1, CNOT6 in the same samples. (B) Quantification of the NDRG1 and MGMT protein expression after each transfection shown as mean ± SEM normalized to controls for six independent experiments; the (*) indicates statistical significance.

To see whether CNOT3 or CNOT6L might be involved in mTORC1 regulation of NDRG1 and MGMT protein expression, we transfected mouse fibroblasts with constructs overexpressing human CNOT3 and CNOT6L (GFP‐CNOT3, GFP‐CNOT6L). Human CNOT3 and CNOT6L share 95–99% of their amino acid sequence with their mouse homologs and thus are likely to function in mouse cells. In addition, we tested the effects of reduction of CNOT3 and CNOT6L expression using silencing constructs (shRNAs) tailored to their respective mouse genes. Mouse fibroblasts were transfected, and efficiency was measured using green fluorescent protein (Fig. S4A, Supporting information). Quantification showed a range from 10 to 30% efficiency, depending on the construct (data not shown). Immunoblotting shows the expected increases or declines in CNOT3 and CNOT6L protein at 72 h after transfection (Fig. S4B, Supporting information). Overexpression of CNOT3 led to reduction in NDRG1 and MGMT protein expression, while CNOT3 downregulation increased NDRG1 and MGMT (Fig. 6A, top panel). Statistical analysis confirmed significant effects in NDRG1 and MGMT expression by CNOT3 (Fig. 6B). Neither overexpression nor reduction of CNOT6L protein affected NDRG1 or MGMT protein expression. Furthermore, changes in CNOT3 and CNOT6L did not affect mTOR activity (no changes in phosphorylation of S6 or AKT_(473)_), suggesting that CCR4‐NOT is downstream of mTOR in regulating NDRG1 and MGMT expression (Fig. 6A). These results suggest that CNOT3 is a post‐transcriptional regulator of NDRG1 and MGMT protein expression and that declines in CNOT3 in Snell, GHRKO, and PAPPA‐KO mice may be involved in the enhanced expression of these DNA repair enzymes.

## Discussion

Our data showing reduction of mTORC1 in multiple tissues of Snell, GHRKO (Dominick *et al*., 2015), and now PAPPA‐KO mice support the hypothesis that mTORC1 controls some of the intracellular events leading to lifespan extension in mammals. It is still unclear, however, how lower mTORC1 action might lead to improved health and better survival in aging mice. Many lines of evidence (Lombard & Miller, 2012) suggest that increases in stress resistance (Murakami, 2006), including better DNA repair (De Luca *et al*., 2013), may be involved in longevity and disease resistance. This idea is now supported by our observation of increases in MGMT and NDRG1, which are involved in direct reversal of DNA damage, in multiple tissues of three stocks of slow‐aging mice. Our evidence that reducing mTORC1 activity leads to enhancement in MGMT and NDRG1 protein expression, both in cultured cells and in mice, strongly suggests a link between mTOR signaling and the DNA repair pathway (Strozyk & Kulms, 2013) that may be central in delaying the aging process. Our data also suggest a possible involvement of mTORC1‐mediated alteration in post‐transcriptional regulation of MGMT and NDRG1 translation via changes in elements of the CCR4‐CNOT complex.

Comparative microarray analysis among several genetic models (Svensson *et al*., 2011; Hofmann *et al*., 2015), pharmacological interventions (Miller *et al*., 2014; Yu *et al*., 2015), and dietary interventions (Bartke *et al*., 2007) known to extend lifespan has not revealed consistent shared features at the level of transcriptional changes, including mRNA levels for enzymes from the DNA repair response such as MGMT and NDRG1. These observations are consistent with our qRT–PCR analysis, which showed no significant changes in NDRG1 and MGMT mRNA expression in the Snell, GHRKO, and PAPPA‐KO mice with respect to controls (Fig. 2). The discrepancy between the mRNA and protein levels suggested that a post‐transcriptional mechanism may be involved in the physiology of these mouse models and in the control of longevity and age‐dependent diseases.

Our data suggest that the CCR4‐NOT complex, a key regulator of post‐transcriptional gene expression, may link mTOR changes to DNA repair in these slow‐aging mice. Snell, GHRKO, and PAPPA‐KO mice show a consistent pattern of changes in CNOT1, CNOT3, CNOT6, and CNOT6L, both in cultured cells and in intact mice, a pattern recapitulated in control cells and in control mice by short‐term exposure to rapamycin. Thus, regulation of key CCR4‐NOT expression components seems to be under the control of mTORC1 (such as CNOT1, CNOT3 and CNOT6L shown in Fig. 5), and down regulation of CNOT3 expression seems to upregulate NDRG1 and MGMT protein expression (Fig. 6). The CCR4‐NOT complex has been studied in yeast (Shirai *et al*., 2014), but little is known about the function of individual components in higher organisms such as mice (Shirai *et al*., 2014).

CCR4‐NOT components, including CNOT3, CNOT6, and CNOT6L, can regulate mRNA stability, microRNA function, localization to the P/Stress bodies, and preferential translation (Shirai *et al*., 2014). However, this regulation involves the interaction of a wide range of tissue‐specific mRNA‐binding proteins (Reese, 2013; Temme *et al*., 2014), and thus more multifaceted work will be needed to determine how the changes in CNOT protein levels shown in Fig. 4 contribute to protection against age‐related changes in specific cell and tissue types. Recent studies in Snell, GHRKO, and PAPPA‐KO mice have shown a common physiology that may control aging rate and multiple age‐related diseases (Murakami *et al*., 2003; Boylston *et al*., 2006; Brown‐Borg, 2006). These mice all exhibit high insulin sensitivity and an increased oxygen consumption with high levels of peroxisome proliferator‐associated receptor γ coactivator 1 (Pgc1α) from oxidative phosphorylation (Corton & Brown‐Borg, 2005; Stauber *et al*., 2005). Similarly, CNOT3 heterozygous mice (Morita *et al*., 2011) have been shown to have increased insulin sensitivity, oxidative phosphorylation (including higher Pgc1α expression), and oxygen consumption. Our data suggest that CCR4‐NOT complex, including CNOT3, could be one of the signaling pathways downstream of mTORC1 regulating some of these changes (Fig. 2). Therefore, it would be interesting to test if declines in CNOT3 [such as in the CNOT3 heterozygous mice (Morita *et al*., 2011)], along with changes in other components of the CCR4‐NOT, could themselves extend lifespan. It would also be interesting to define what specific mechanism the CCR4‐NOT components are using to enhance expression of the MGMT and NDRG1 DNA repair genes, such as regulation by microRNA or preferential translation (or both). Because many of the CCR4‐NOT components are essential for development, future studies of CCR4‐NOT and its effects on DNA repair and the aging process would require the use of conditional knockouts triggered in adulthood. Of particular interest in the aging processes would be CCR4‐NOT components that affect post‐transcriptional mechanisms, such as CNOT6 and CNOT6L. Alternatively, when available, the analysis of CNOT6 and CNOT6L +/− heterozygous mice would also shed some light on possible functions of CNOT in aging. On the other hand, it is also possible that changes in proteasomal function in slow‐aging mice could contribute to the elevation of MGMT, NDRG1, and CNOT6L (Wang & Miller, 2012; Zhang *et al*., 2014; Zhang & Manning, 2015).

Increases in CNOT6L have been suggested to increase the ATM‐dependent DNA repair response (Sanchez‐Perez *et al*., 2009). In this context, it will be interesting to determine whether tissues from slow‐aging mice have increases in other DNA repair mechanisms, or, alternatively, whether being heterozygous for CNOT components would lead to changes in DNA repair that may affect the aging process. Overall, the data presented here suggest a signaling pathway in which alterations in mTOR, including declines in mTORC1, lead to alterations in post‐transcriptional regulation of gene expression, including genes involved in DNA repair, via changes in composition and function of the CCR4‐NOT complex.

## Material and methods

### Reagents, mice, and fasting and feeding conditions

Snell (DW) were produced as previously described (Dominick *et al*., 2015). Breeding stock for GHRKO mice was provided by Andrzej Bartke (Southern Illinois University, IL) and John Kopchick (Edison Biotechnology Institute of Ohio University, OH) and mice were produced as previously described (Dominick *et al*., 2015). Breeding stock for PAPPA‐KO mice was provided by Cheryl Conover (Mayo Clinic, Rochester, MN) and mice were produced as previously described (Conover *et al*., 2004). DW, GHRKO, and PAPPA‐KO mice were used at ages from 5 to 6 months. Genetically heterogeneous mice (UM‐HET3) ranging from 10 to 12 months of age were produced by a cross between two different F1 parents: (BALB/cByJ × C57BL/6J)F1 mothers and (C3H/HeJ × DBA/2J)F1 fathers as previously described (Harrison *et al*., 2009). All experiments were approved by the University of Michigan University Committee on Use and Care of Animals. Sentinel animals were examined quarterly for evidence of viral infection; all tests were negative. Mice used were fed ad lib or, as indicated in the text, fasted for 18 h (fasted mice) followed by ad lib feeding with a regular chow diet for 6 h (fed mice). All mice had free access to water during the course of treatments. Chemical reagents were purchased from Sigma (www.sigmaldrich.com). Rapamycin was purchased from LC laboratories (www.lclabs.com). The source of antibodies and phospho‐specific antibodies are described in Table S3. Primary mouse fibroblasts from UM‐HET3 mice were cultured as described (Murakami *et al*., 2003). Clones corresponding to human CNOT3 (pT7‐EGFP‐HsNOT3, Addgene plasmid 37369) and CNOT6L (pT7‐EGFP‐HsNOT6L, Addgene plasmid 37372) were a gift from Elisa Izaurralde (Max Planck Institute for Developmental Biology, Germany). The shRNA for mouse CNOT3 (NM_146176.2) and mouse CNOT6L (NM_144910.1) were purchased from Genecopoeia (www.genecopoeia.com). Plasmid transfections were carried out with lipofectamine 3000 as described by the manufacturer (www.thermofisher.com). qRT–PCR was performed as described (Li *et al*., 2013). Probes were manufactured by IDT (www.idtdna.com) corresponding to the sequences shown in Table S4.

### Rapamycin treatments, tissue preparation, Western blot analysis

Acute rapamycin treatments in UM‐HET3 mouse fibroblasts were performed at 0.1 μm. *In vivo* rapamycin treatments were performed with a single intraperitoneal injection of rapamycin at 5 mg kg^−1^ to each UM‐HET mouse (ranging from 10 to 12 months of age) as previously described (Houde *et al*., 2010). Tissues from each mouse were collected, frozen with liquid nitrogen, and stored at −70 °C. The tissue samples were then processed to obtain cell lyses for Western blots (Dominick *et al*., 2015) or qRT–PCR analysis (Li *et al*., 2013). Mouse fibroblasts were collected and lysed, and equal amounts of protein were loaded for Western blot analysis (Murakami *et al*., 2003).

### Statistical analysis

Unless indicated otherwise, results are presented as mean ± standard error of the mean (SEM). Statistical tests for genotype, fasting and fed conditions (nutritional), or interaction effects were assessed by two‐way analysis of variance (ANOVA) using *P* = 0.05 as the threshold for significant effects. To compare specific effects between groups, an unpaired t‐test was used with *P* = 0.05 as the criterion for significance.

## Funding

This work was supported by grants from the National Institutes of Health AG019899 and AG024824 and the University of Michigan Paul Glenn Center for the Biology of Aging. The funders had no role in study design, data collection and analysis, decision to publish, or preparation of the manuscript.

## Conflict of interest

The authors have declared that no conflict of interest exist.

## Supporting information


**Fig. S1** The CT dotplot graphs for GAPDH mRNA used for normalization controls from liver samples from Snell (DW), GHRKO (B) or PAPPA (C) show no statistically significant differences.
**Fig. S2** The CT dotplot graphs for CNOT4 and GAPDH mRNA used as normalizing controls from liver samples of Snell (A) and GHRKO (B) show no statistically significant differences.
**Fig. S3** The CT value graphs representing the CNOT mRNA levels in mouse fibroblasts untreated (−, control) or treated with Rapamycin (+, Rapa) from 6 independent experiments. The (*) indicates statistical significance.
**Fig. S4** (A) Representative phase contrast and green fluorescence channels of mouse fibroblasts overexpressing the human GFP‐CNOT3 or GFP‐CNOT6L, as well as the silencing shCNOT3, shCNOT6L and respective control vectors.
**Table S1** Effects of genotype and feeding on the ratio of pS6 and p4E‐BP1 in different tissues from PAPP‐A (KO) mice compared to control mice (WT).
**Table S2** Effects of genotype and feeding on the ratio of pNDRG1 and pAKT(450) in different tissues from PAPP‐A (KO) mice compared to control mice (WT).
**Table S3** Source of antibodies.
**Table S4** qRT‐PCR probes.
