# Supplementary material for: mTOR regulates the expression of DNA damage response enzymes in long‐lived Snell dwarf, GHRKO, and PAPPA‐KO mice
Source: Aging Cell. 2016 Sep 13;16(1):52–60. doi: 10.1111/acel.12525 (PMC5242303; doi:10.1111/acel.12525)
Supplement: Supplementary file 1 — Fig. S1 The CT dotplot graphs for GAPDH mRNA used for normalization controls from liver samples from Snell (DW), GHRKO (B) or PAPPA (C) show no statistically significant differences. Fig. S2 The CT dotplot graphs for CNOT4 and GAPDH mRNA used as normalizing controls from liver samples of Snell (A) and GHRKO (B) show no statistically significant differences. Fig. S3 The CT value graphs representing the CNOT mRNA levels in mouse fibroblasts untreated (−, control) or treated with Rapamycin (+, Rapa) from 6 independent experiments. The (*) indicates statistical significance. Fig. S4 (A) Representative phase contrast and green fluorescence channels of mouse fibroblasts overexpressing the human GFP‐CNOT3 or GFP‐CNOT6L, as well as the silencing shCNOT3, shCNOT6L and respective control vectors. Table S1 Effects of genotype and feeding on the ratio of pS6 and p4E‐BP1 in different tissues from PAPP‐A (KO) mice compared to control mice (WT). Table S2 Effects of genotype and feeding on the ratio of pNDRG1 and pAKT(450) in different tissues from PAPP‐A (KO) mice compared to control mice (WT). Table S3 Source of antibodies. Table S4 qRT‐PCR probes. [file ACEL-16-52-s001.docx]

**Table 1. Effects of genotype and feeding on the ratio of pS6 and p4E-BP1 in different tissues from PAPP-A (KO) mice compared to control mice (WT).** Values represent the mean fold change between compared groups. Unpaired t-tests were used on untransformed data to compare individual groups. *p < 0.05, **p < 0.01, ***p < 0.001. Two-way ANOVA was used to test the genotype (G, p < 0.05), nutritional (N, p < 0.05) effects using normalized values as described in Figure 1.

| **Tissue** | **Protein** | **WT/KO**  **(Fasted)** | **WT/KO**  **(Fed)** | **Fed/Fasted**  **(KO)** | **Fed/Fasted**  **(WT)** | **ANOVA** |
| --- | --- | --- | --- | --- | --- | --- |
| **LIVER** | **pS6** | 2.1** | 1.8** | 3.9*** | 3.3** | G+N |
|  | **p4E-BP1** | 1.6*** | 1.6** | 2.1*** | 2.0** | G+N |
| **SKELETAL MUSCLE** | **pS6** | 2.5* | 1.5* | 4.6*** | 2.8** | G+N |
|  | **p4E-BP1** | 1.5* | 1.3 | 1.7* | 1.4* | G+N |
| **HEART** | **pS6** | 3.0* | 1.3*** | 4.7** | 2.0* | G+N |
|  | **p4E-BP1** | 1.5* | 1.6* | 1.6** | 2.0* | G+N |
| **KIDNEYS** | **pS6** | 2.3* | 2.3* | 2.4* | 2.7* | G+N |
|  | **p4E-BP1** | 2.2 | 2.6* | 1.8 | 2.5* | G+N |

**Table 2. Effects of genotype and feeding on the ratio of pNDRG1 and pAKT(450) in different tissues from PAPP-A (KO) mice compared to control mice (WT).** Values represent the mean fold change between compared groups. Unpaired t-tests were used on untransformed data to compare individual groups. *p < 0.05, **p < 0.01, ***p < 0.001. Two-way ANOVA was used to test the genotype (G, p < 0.05), nutritional (N, p < 0.05) and interaction (Int, p < 0.05) effects using of normalized values as described in Figure 1.

| **Tissue** | **Protein** | **WT/KO**  **(Fasted)** | **WT/KO**  **(Fed)** | **Fed/Fasted**  **(KO)** | **Fed/Fasted**  **(WT)** | **ANOVA** |
| --- | --- | --- | --- | --- | --- | --- |
| **LIVER** | **pNDRG1** | 0.6** | 1.1 | 0.8** | 1.4* | G+Int |
|  | **pAKT(450)** | 0.5* | 1.0 | 0.6* | 1.5* | G+Int |
| **SKELETAL MUSCLE** | **pNDRG1** | 0.7** | 1.2 | 0.6*** | 1.0 | G+N+Int |
|  | **pAKT(450)** | 0.5* | 1.2 | 0.5* | 1.2 | G+Int |
| **HEART** | **pNDRG1** | 0.6** | 1.1 | 0.8** | 1.1 | G+N+Int |
|  | **pAKT(450)** | 0.4* | 1.0 | 0.7 | 1.8 | G |
| **KIDNEYS** | **pNDRG1** | 0.7* | 1.2 | 0.6** | 1.1 | G+Int |
|  | **pAKT(450)** | 0.6* | 1.1 | 0.5* | 1.0 | G+N+Int |

**Table 3. Source of Antibodies**

| **Antibody** | **Source** | **Cat #** |
| --- | --- | --- |
| **pS6(235)** | Cell Signaling | 2211 |
| **S6** | Cell Signaling | 2217 |
| **p4E-BP1(T36/46)** | Cell Signaling | 2855 |
| **4E-BP1** | Cell Signaling | 9644 |
| **pAKT(473)** | Cell Signaling | 4060 |
| **pAKT(450)** | Cell Signaling | 12178 |
| **AKT** | Cell Signaling | 9272 |
| **pNDRG1(T346)** | Cell Signaling | 5482 |
| **NDRG1** | Cell Signaling | 9408 |
| **MGMT** | MyBioSource | 9409314 |
| **ACTIN** | Santa Cruz | 47778-HRP |
| **CNOT-1** | LSBio | 335614 |
| **CNOT-3** | Aviva Systems | 34318 |
| **CNOT-6** | Santa Cruz | 368367 |
| **CNOT-6L** | Santa Cruz | 246304 |

**Table 4. qRT-PCR probes.**

| **Probe** |  | **Squence** |
| --- | --- | --- |
| **MGMT** | Forward | aaacactgaccccacagagg |
|  | Reverse | aacacagggtgatggagagc |
| **NDRG1** | Forward | cgagagctacatgacgtgga |
|  | Reverse | aagagggggttgtagcaggt |
| **CNOT1** | Forward | tctctgcgtttgttggacag |
|  | Reverse | aggcatccaggttgtggtag |
| **CNOT3** | Forward | gtctgggtctggctcagaag |
|  | Reverse | tcaatccggtcctgcttatc |
| **CNOT4** | Forward | agtgcgaggtctccttttga |
|  | Reverse | gaggctctgtggttctggag |
| **CNOT6** | Forward | ttgccaagcttcacaatctg |
|  | Reverse | tctggctccagacagaggtt |
| **CNOT6L** | Forward | cgggtgttgccttatgaact |
|  | Reverse | ggaagctgctctggatgaac |

**Supplemental Figure 1.** The CT dotplot graphs for GAPDH mRNA used for normalization controls from liver samples from Snell (DW), GHRKO (B) or PAPPA (C) shows no statistical significant differences.

**
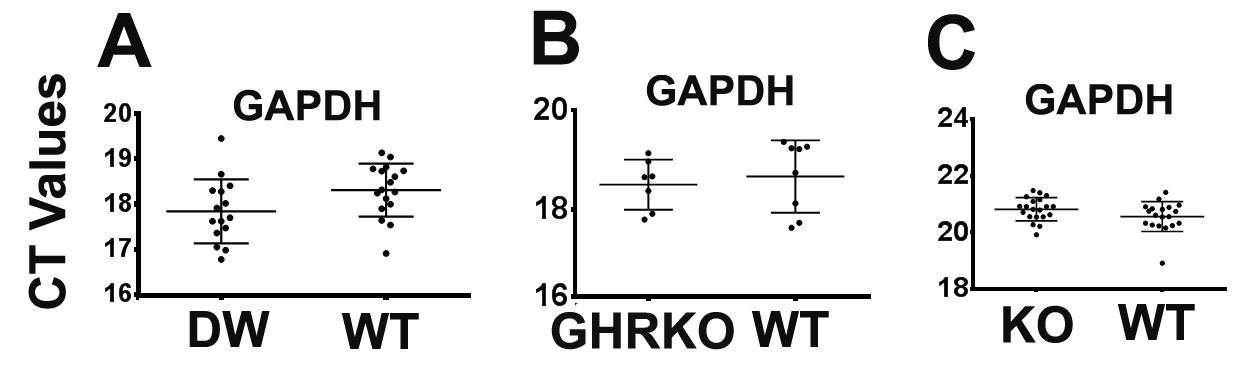
**

**Supplemental Figure 2.** The CT dotplot graphs for CNOT4 and GAPDH mRNA used is normalizing controls from liver samples of Snell (A) and GHRKO (B) shows no statistical significant differences.

**
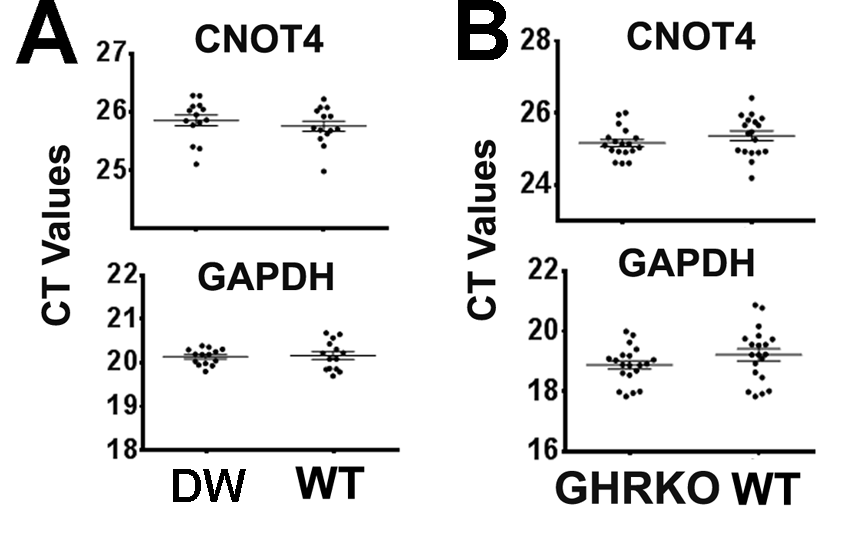
**

**Supplemental Figure 3.** The CT value graphs represent the CNOT mRNA levels in mouse fibroblasts untreated (- , control) or treated with Rapamycin ( **+** , Rapa) from 6 independent experiments. The (*) indicates statistical significance.

**
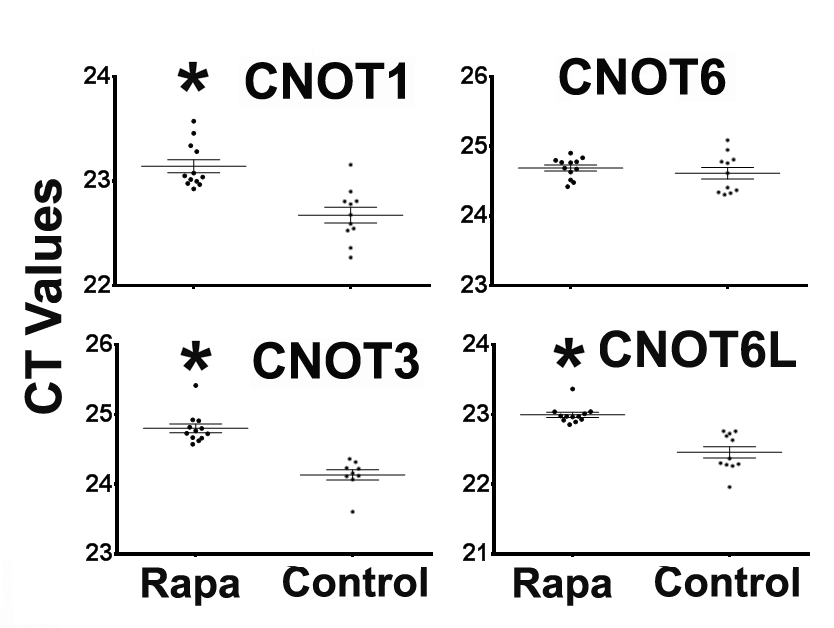
**

**Supplemental Figure 4. A)** Representative phase contrast and green fluorescence channels of mouse fibroblasts overexpressing the human GFP-CNOT3 or GFP-CNOT6L, as well as the silencing shCNOT3, shCNOT6L and respective control vectors. **B)** Representative western blots against CNOT3 and CNOT6L (endogenous and GFP-tag proteins) in mouse fibroblasts transfected with these vectors.

**
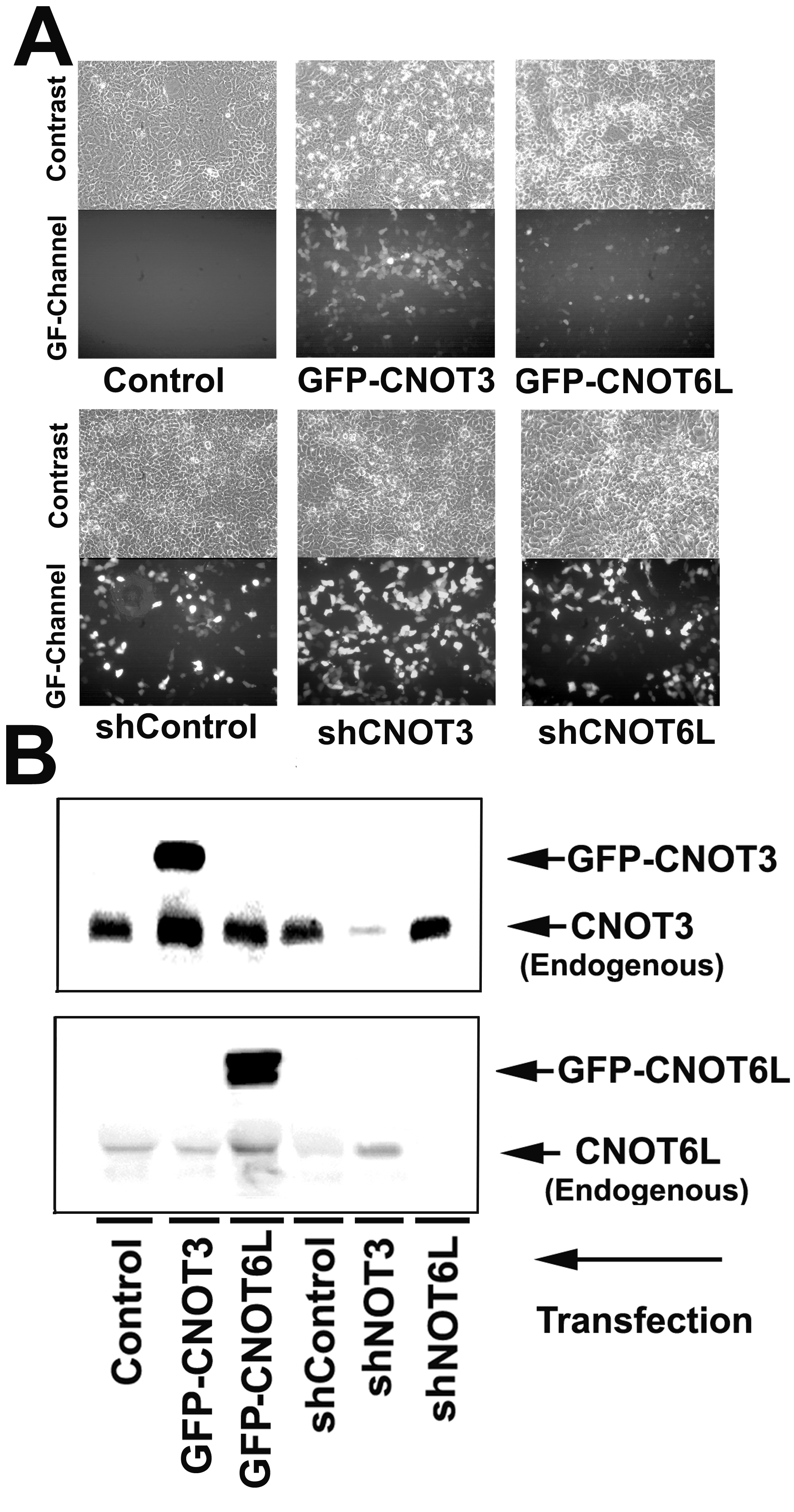
**
